# Radiographic Assessment of Spinal Degeneration in Vervet Monkeys (*Chlorocebus aethiops*): Prevalence, Patterns, and Relevance to Primate Aging Models

**DOI:** 10.1002/ajp.70136

**Published:** 2026-03-23

**Authors:** Camille Gabriela Ramos Portal, Aline Amaral Imbeloni, Sheila Canevese Rahal, Wellington Bandeira da Silva, Washington Takashi Kano, Seizo Yamashita, Pedro Mayor, Frederico Ozanan Barros Monteiro

**Affiliations:** ^1^ Graduate Program in Animal Health and Production in the Amazon Federal Rural University of Amazonia (UFRA) Belém Pará Brazil; ^2^ National Primate Center, Health Surveillance Secretariat, Ministry of Health Ananindeua Pará Brazil; ^3^ Department of Veterinary Surgery and Animal Reproduction, School of Veterinary Medicine and Animal Science São Paulo State University (UNESP) Botucatu São Paulo Brazil; ^4^ Non‐Human Primate Breeding Service – Institute of Science and Technology in Biomodels (ICTB), Fiocruz Rio de Janeiro Rio de Janeiro Brazil; ^5^ Medical School São Paulo State University (UNESP) Botucatu São Paulo Brazil; ^6^ Departament d'Anatomia i Salut Animal Universitat Autònoma de Barcelona, Bellaterra Barcelona Spain

**Keywords:** animal welfare, laboratory animals, musculoskeletal aging, nonhuman primates, osteophytes, translational model

## Abstract

Degenerative changes of the vertebral column are common in aging primates; however, patterns of spinal osteopathy remain poorly characterized across small‐ and medium‐bodied primate species, limiting comparative interpretations of skeletal aging. Expanding assessments beyond well‐studied taxa provides a broader framework for understanding primate musculoskeletal senescence and generates baseline data essential for interpreting vertebral degeneration in wild and semi‐free‐ranging populations, where ecological and life‐history factors influence skeletal aging. We investigated age‐, sex‐, and body mass–related variation in vertebral osteopathies radiographically assessed in 70 captive vervet monkeys (*Chlorocebus aethiops*). Osteophytes were the most frequent lesion (78.6%), followed by discopathy (12.7%), syndesmophytes (2.9%), and scoliosis (1.4%). Age was the primary predictor of osteophyte presence across spinal regions, whereas sex and body mass showed no significant independent effects. In contrast, total osteophyte scores were significantly higher in older, heavier individuals, and females exhibited slightly higher adjusted scores than males. Nonlinear regression models revealed distinct age‐related trajectories among spinal regions, with osteophytes emerging earliest in the lumbar spine (~ 1 year), followed by the cervical (~ 7 years) and thoracic (~ 9 years) regions. These findings characterize the natural history of spinal degeneration in vervet monkeys under captive conditions and provide comparative baseline data for distinguishing age‐related changes from pathological alterations in both captive and free‐ranging primate populations.

## Summary

- Radiographic assessment of 70 vervet monkeys maintained under human care revealed a high prevalence of age‐related spinal degeneration, with osteophytes predominantly affecting the lumbar spine.
- Age was the strongest predictor of osteophyte presence and cumulative burden, with region‐specific trajectories showing earliest onset in the lumbar vertebrae.
- Findings provide comparative baseline data for interpreting spinal aging in primates and support *Chlorocebus aethiops* as a relevant model for musculoskeletal senescence research.

## Graphical abstract

Radiographic evaluation of 70 vervet monkeys (*Chlorocebus aethiops*) maintained under human care revealed a high prevalence of age‐related spinal degeneration. Osteophytes emerged earliest and most prominently in the lumbar region. These region‐specific patterns parallel those reported in other catarrhine primates, supporting *C. aethiops* as a comparative model for spinal aging.

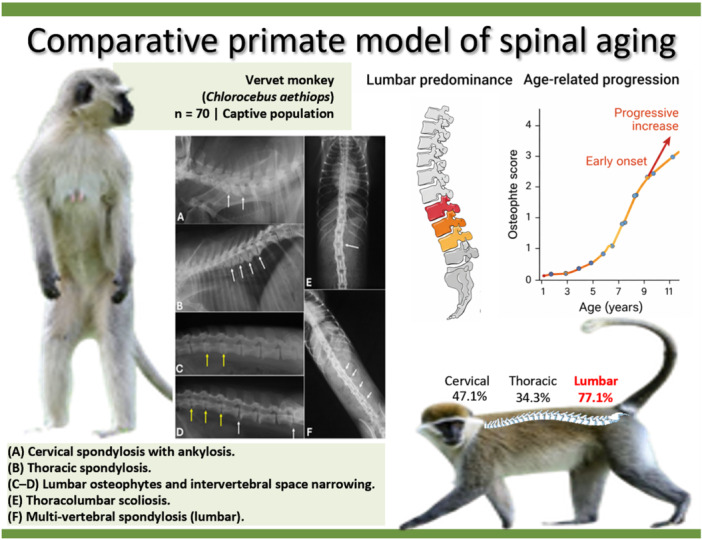

## 1 Introduction

Degenerative diseases of the vertebral column are a major cause of morbidity in humans, and laboratory animal models are essential to advance their understanding and management. Despite postural differences, the spinal column of nonhuman primates (NHPs) is morphologically comparable to that of humans, reinforcing their value in translational studies of spinal health (Pritzker and Kessler 2012). NHPs have long been used to investigate diverse human conditions, from reproductive to musculoskeletal disorders, highlighting their importance in comparative medicine. Notably, studies in female squirrel monkeys (*Saimiri* sp.) showed that, despite anatomical differences, similarities in pelvic support and age‐related changes further support the translational relevance of primates (Pereira da Silva et al. 2021). In addition, the study of degenerative diseases of the vertebral column is essential for the better understanding of competence restrictions of aging individuals from NHP free‐ranging populations.

In both humans and NHPs, the spine may be affected by a wide range of disorders, including structural/postural alterations, degenerative and inflammatory processes, congenital anomalies, trauma, metabolic disturbances, and neoplasia (Kramer et al. 2002). Among these, degenerative changes such as osteopenia, osteophytes, spondylosis, and osteoporosis are especially relevant, as they have been reported in NHPs and represent important models of chronic disability (Cerroni et al. 2000; Hernández‐Godínez et al. 2010; Schmidt 2011).

For laboratory animals, radiographic assessment provides a cost‐effective and widely accessible diagnostic approach, enabling the identification and staging of spinal changes, and supporting both clinical decision‐making and welfare monitoring. Although advanced modalities such as computed tomography and magnetic resonance imaging provide greater diagnostic accuracy (Ruiz Santiago et al. 2022), conventional radiography remains a fundamental tool in laboratory settings due to its practicality and reproducibility (Li and Yao 2015). Radiological findings of degenerative disease, such as narrowing of the intervertebral space, endplate sclerosis, spondylophytes, and joint degeneration, have been consistently documented in humans and NHPs (Schmidt 2011).

Age‐related vertebral degeneration has been extensively documented in Old World monkeys, particularly *Macaca mulatta* and *Papio* spp., where osteophyte formation, disc space narrowing, and endplate remodeling increase predictably with age (Grynpas et al. 1993; Bailey et al. 2014; Nuckley et al. 2008). These species therefore provide well‐characterized comparative frameworks for interpreting degenerative patterns in other primates. However, despite the extensive literature in macaques and baboons, vertebral degeneration in *Chlorocebus* spp., including *C. aethiops*, remains poorly documented, underscoring the need for baseline data on spinal aging in this genus, particularly in a medium‐bodied Old World primate, underscoring the need for baseline data on spinal aging in this genus.

The vervet monkey (*Chlorocebus aethiops*) is an Old‐World species belonging to the family Cercopithecidae and originally native to East Africa (Butynski and Jong 2022). The genus *Chlorocebus*, formerly grouped within *Cercopithecus*, is now recognized as an independent taxon comprising several closely related species (Dessalegn 2019). Within *C. aethiops*, four subspecies are described: *C. a. aethiops*, *C. a. hilgerti*, *C. a. matschiei*, and *C. a. ellenbecki*. Adult vervet monkeys exhibit moderate sexual dimorphism, with males generally presenting greater body mass than females, and body weight increasing until early adulthood before stabilizing (Turner et al. 1997; Butynski and Jong 2022). Under human care, this species reaches an extended lifespan compared to free‐ranging populations, allowing the expression of age‐related musculoskeletal changes, including degenerative alterations of the vertebral column (Rothschild and Woods 1992).

The present study radiologically evaluates and characterizes age‐related degenerative changes of the vertebral column in *C. aethiops* maintained under laboratory conditions. Specifically, we quantify the prevalence and regional distribution of osteophytes and other radiographic findings, and examine their associations with age, body mass, and sex. Finally, we discuss the implications of these results for animal welfare, colony management, and the implications of these findings for animal welfare, colony management, and the interpretation of age‐related spinal degeneration in primate populations.

## 2 Materials and Methods

### 2.1 Subjects and Husbandry

We conducted a radiographic analysis in 70 vervet monkeys (*Chlorocebus aethiops*) maintained at the National Primate Center (CENP, Ananindeua, Pará, Brazil). The study population comprised 36 females and 34 males, with a mean age of approximately 12.2 years (range: 1–25 years), with body masses ranging from 1.27 to 6.65 kg.

Animals were maintained in social groups consisting of a maximum of seven individuals. They were kept in enclosures constructed of brick and covered with tiles and wire netting. The cages measured 3 m in length, 3 m in width, and 3 m in height. The grounds had a double lateral communication‐controlled sliding door, which was used for managing the group. These enclosures were positioned in a north–south orientation to receive 12 h of natural light, providing the animals with regular access to sunlight throughout the year. The climate was classified as humid mesothermal, with average rainfall of 242 mm from January to June. The average temperature was 27°C (max. 32°C and min. 22°C), with a relative humidity of approximately 82% (Instituto Nacional de Meteorologia 2015). Daily care included cleaning of the enclosures and meals offered twice a day: a pelleted feed for primates in the morning (Megazoo P18, Protein 18%, Fiber Max 6.5%, Betim, MG, Brazil), horticultural food in the afternoon, and water ad libitum.

This study adhered to the guidelines of the National Council for the Control of Animal Experimentation (CONCEA) and to the ARRIVE guidelines (Animal Research: Reporting of In Vivo Experiments). It was approved by the Commission for Ethics in Animal Research of the Evandro Chagas Institute (protocol No. 0014/2014‐CEUA‐IEC) at the Evandro Chagas Institute (IEC), Ananindeua, Pará, Brazil, and by the Biodiversity Authorization and Information System of the Chico Mendes Institute of Biodiversity (Sisbio/ICMBio, protocol 38529).

### 2.2 Immobilization Methods and Radiographic Analysis

The vervet monkeys were chemically restrained using an anesthetic protocol adapted from Hernández‐Godínez et al. (2010), consisting of 4 mg/kg IM of tiletamine hydrochloride (125 mg) combined with zolazepam hydrochloride (125 mg), diluted in distilled water (5 mL) (Zoletil 50, Virbac, Brazil). All animals were weighed with a Filizola MF‐30 digital scale (Indústrias Filizola S/A, Rua Joaquim Carlos, São Paulo, SP, Brazil). Heart rate, temperature, and respiratory rate were monitored throughout the procedure, and after full anesthetic recovery the animals were returned to their original social group.

Plain spinal radiographs were performed in ventrodorsal, right lateral, and left lateral positions, including the cervical, thoracic, lumbar, and sacral regions (C1–S5). A focal film distance of 100 cm was used, with an exposure of 70 kV, 100 mA, and 6.4 mAs (Intercal Cr‐7, 100 mAs, 90 kV).

All radiographs were evaluated for congenital, inflammatory, neoplastic, degenerative, and traumatic lesions by a single experienced radiologist. Osteophytes were classified using a qualitative ordinal scale based on their radiographic appearance, rather than on direct metric measurements. The scoring criteria were: (0) absent, when no cortical projection was visible; (1) mild, when small, focal, well‐defined bony protrusions were present without bridging or marked deformation of the vertebral margins; and (2) severe, when large osteophytes caused evident contour irregularity, partial bridging, or advanced endplate remodeling. Osteophytes were recorded separately for each vertebral region (cervical, thoracic, lumbar, and sacral), and the total osteophyte burden per animal was calculated by summing the regional scores.

### 2.3 Statistical Analysis

The occurrence of osteophytes in each vertebral region and in any region was analyzed using generalized linear models (GLMs) with binomial distribution and logit link function, including sex, age, and body mass as explanatory variables. The sacral region was excluded from the regional analyses because no osteophytes were observed in this segment. The total osteophyte score per individual was analyzed using a GLM with Poisson distribution and log link function, with sex, age, and body mass as predictors.

The relationship between age and body mass was evaluated using nonlinear regression, and the best‐fitting models for age‐related changes in osteophyte development were selected based on the coefficient of determination (*R*²). Differences in body mass between sexes were tested using Student's *t*‐test.

All statistical analyses were performed using Jamovi, GraphPad Prism, and CurveExpert Professional. A significant level of *p* < 0.05 was adopted. Data are presented as mean ± standard deviation (SD). The full results of the GLMs are provided in the Supplementary Tables.

## 3 Results

**FIGURE 1:**
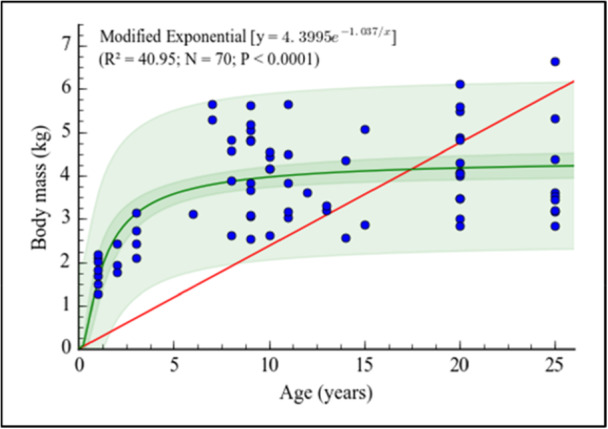
Relationship between body mass and age in the *Chlorocebus aethiops* (*n* = 70). The green line represents the best model fitted to the plots ± 95% CI, red line represents an expected linear trend with no intercept.

Females had a mean age of 11.6 ± 7.7 years (range: 1–25 years) and a mean body mass of 3.03 ± 0.73 kg (range: 1.69–4.48 kg). Males showed a comparable mean age of 12.8 ± 7.9 years (range: 1–25 years), but a significantly higher mean body mass of 4.38 ± 1.29 kg (range: 1.27–6.65 kg). Age dispersion was similar between sexes, whereas body mass exhibited greater variability and higher values in males. Body mass increased significantly with age and was best described by a modified exponential model (*R*² = 0.4095, *p* < 0.0001; Figure 1). Males were significantly heavier than females (*p* < 0.0001).

**FIGURE 2:**
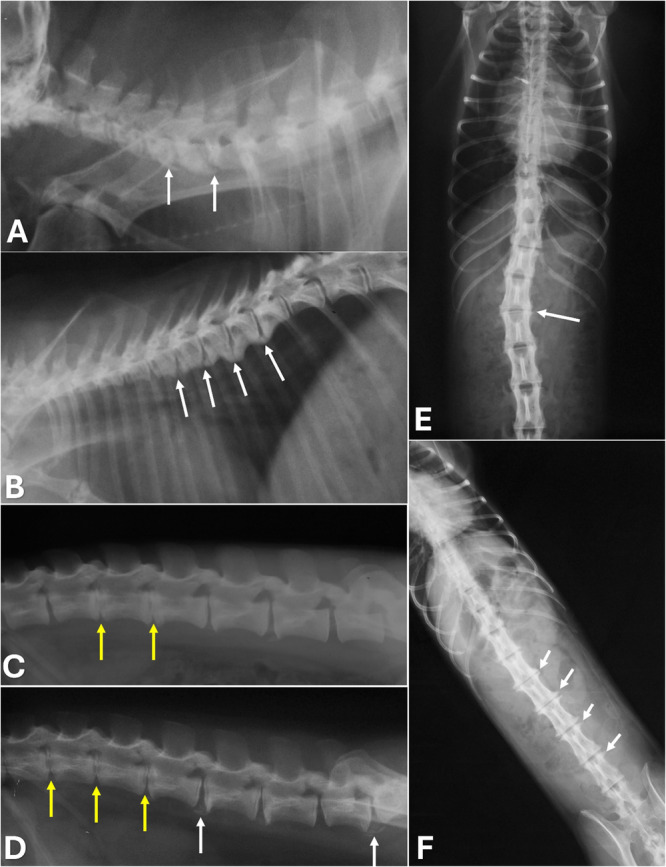
Radiographic images of the vertebral column in the *Chlorocebus aethiops*. (A) Cervical segment, lateral view: ventral spondylosis with ankylosis tendency (arrows). (B) Thoracic segment: ankylosing ventral spondyloses (arrows). (C) Lumbar spine, lateral view: reduced intervertebral spaces (yellow arrows) and mild spondylosis (white arrows). (D) Lateral view: endplate sclerosis and narrowed intervertebral spaces (yellow arrows). (E) Ventrodorsal view: thoracolumbar scoliosis (arrow). (F) Ventrodorsal view: lateral spondylosis in multiple lumbar vertebrae (arrows).

Osteophytes were the most frequent osteopathy (78.6%; 55/70), followed by discopathy (12.7%, 9/70), syndesmophytes (2.9%, 2/70), and scoliosis (1.4%, 1/70) (Figure 2). Osteophytes were observed in 80.6% (29/36) of females and 76.5% (26/34) of males (Table 1). Regionally, the lumbar spine showed the highest prevalence (77.1%), followed by the cervical (47.1%) and thoracic (34.3%) regions, while no osteophytes were detected in the sacral vertebrae. The lumbar region also exhibited the highest proportion of mild (44.3%) and severe (32.9%) lesions (Table 2).

**TABLE 1:** Absolute and relative (%) frequencies of osteophytes, categorized by sex, according to spine region in 36 females and 34 males *Chlorocebus aethiops* (*n* = 70).

Spine regions	Females	Males	Females and males
Cervical	15 (41.7%)	18 (52.9%)	33 (47.1%)
Thoracic	13 (36.1%)	11 (32.3%)	24 (34.3%)
Lumbar	29 (80.5%)	25 (73.5%)	54 (77.1%)
Sacral	0 (0.0%)	0 (0.0%)	0 (0.0%)
Any region	29 (80.5%)	26 (76.5%)	55 (78.6%)

**TABLE 2:** Absolute and relative (%) osteophytes frequencies in each vertebral region in 36 females and 34 males *Chlorocebus aethiops* (*n* = 70).

Osteophytes stratification	Sex	Spine region			
		Cervical	Thoracic	Lumbar	Sacral
Absent	♀ (*n* = 36)	21 (58.3%)	23 (63.9%)	7 (19.4%)	36 (100%)
	♂ (*n* = 34)	16 (47.1%)	23 (67.7%)	9 (26.4%)	34 (100%)
	♀ ♂ (*n* = 70)	37 (52.9%)	46 (65.7%)	16 (22.9%)	70 (100%)
Mild	♀ (*n* = 36)	9 (25%)	9 (25.0%)	18 (50.0%)	0 (0%)
	♂ (*n* = 34)	10 (29.41%)	10 (29.4%)	13 (38.2%)	0 (0%)
	♀ ♂ (*n* = 70)	19 (27.14%)	19 (27.1%)	31 (44.3%)	0 (0%)
Severe	♀ (*n* = 36)	6 (16.67%)	4 (11.1%)	11 (30.6%)	0 (0%)
	♂ (*n* = 34)	8 (23.53%)	1 (2.9%)	12 (35.3%)	0 (0%)
	♀ ♂ (*n* = 70)	14 (20%)	5 (7.1%)	23 (32.9%)	0 (0%)

**FIGURE 3:**
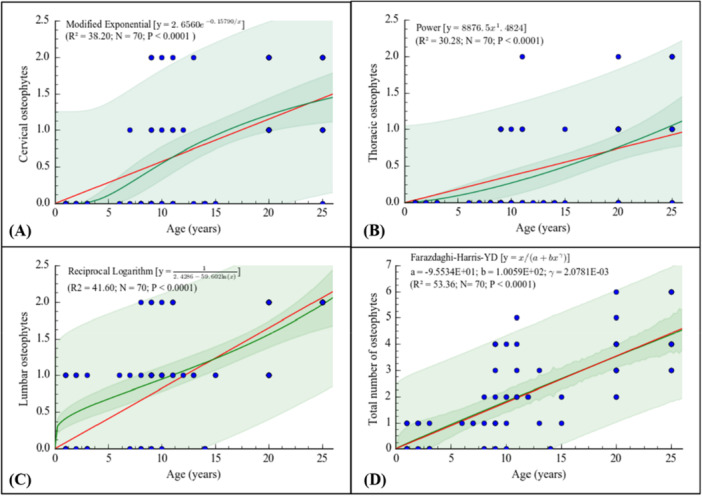
Relationship between the number of osteophytes in cervical (A), thoracic (B), lumbar (C), and total osteophytes (D) in function of the age (years) in the *Chlorocebus aethiops* (*n* = 70). The green line represents the best model fitted to the plots ± 95% CI, red line represents an expected linear trend with no intercept.

The number of osteophytes per vertebral region increased significantly with age (Figure 3). In the cervical spine, a modified exponential model (*R*² = 38.20) described the age‐related trajectory, with osteophytes emerging at approximately 7 years of age (Figure 3A). In the thoracic region, a power model (*R*² = 30.28) indicated onset around 9 years, with severe changes appearing after approximately 11 years (Figure 3B). The lumbar spine showed the earliest involvement, with a reciprocal logarithmic model (*R*² = 41.60) indicating mild osteophytes from approximately 1 year of age and severe lesions from about 7 years onward (Figure 3C). No osteophytes were observed in the sacral region.

Binomial GLMs confirmed that age was the principal predictor of osteophyte presence (Supporting Information Table S1). In the cervical spine, age (*β* = 0.273 ± 0.072, *z* = 3.78, *p* < 0.001) and body mass (*β* = 1.123 ± 0.570, *z* = 1.97, *p* = 0.049) were significantly associated with osteophyte presence, whereas sex was not (*β* = 1.177 ± 1.040, *p* = 0.258). In the thoracic region, age remained significant (*β* = 0.178 ± 0.051, *z* = 3.49, *p* < 0.001), while sex (*p* = 0.058), and body mass (*p* = 0.068) were not statistically significant. In the lumbar spine, age was also positively associated with osteophyte occurrence (*β* = 0.148 ± 0.070, *z* = 2.11, *p* = 0.034), with no independent effects of sex (*p* = 0.121) or body mass (*p* = 0.202). When osteophyte presence was considered in any region, no independent associations with sex (*p* = 0.119), age (*p* = 0.082), or body mass (*p* = 0.080) were detected.

Considering cumulative severity, total osteophyte scores were descriptively similar between females (78 points; 27.1% of the maximum possible score) and males (75 points; 27.6%) (Table 3). However, in the Poisson GLM evaluating total osteophyte burden (Supporting Information Table S2), age was the strongest predictor (*β* = 0.069 ± 0.012, *z* = 5.92, *p* < 0.001). Body mass showed a significant positive association (*β* = 0.254 ± 0.103, *z* = 2.47, *p* = 0.014), and sex exhibited a modest but statistically significant effect (*β* = 0.461 ± 0.234, *z* = 1.97, *p* = 0.049), indicating slightly higher adjusted scores in females after controlling for age and body mass.

**TABLE 3:** Total osteophyte scores by sex in 36 females and 34 males *Chlorocebus aethiops* (*n* = 70).

Group	*N*	Total osteophyte score	% of maximum
Females	36	78	27.1
Males	34	75	27.6
Total	70	153	27.3

**FIGURE 4:**
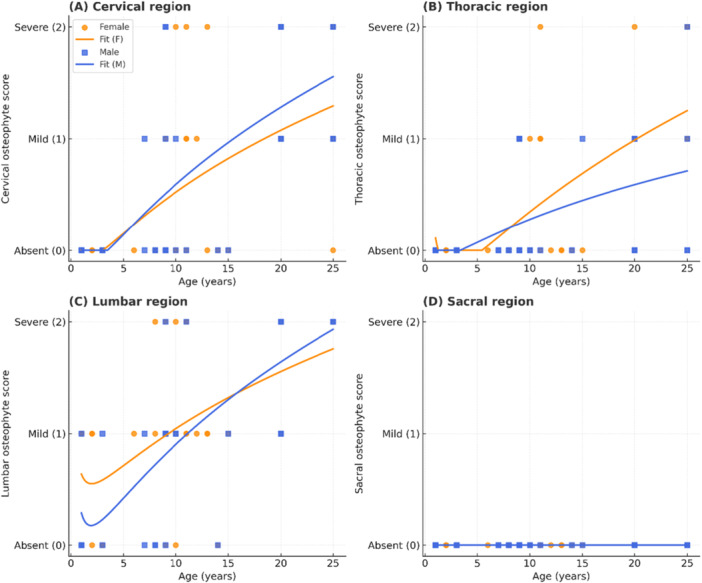
Age‐related distribution of osteophyte scores in *Chlorocebus aethiops* (*n* = 70). Scatter plots show females (orange circles) and males (blue squares), with reciprocal logarithmic fits for females (orange line) and males (blue line). (A) Cervical region; (B) Thoracic region; (C) Lumbar region; (D) Sacral region. Osteophyte scores were classified as absent (0), mild (1), or severe (2). Mild osteophytes were observed from 1 year of age in both sexes, whereas severe lesions appeared from 8 years in females and 9 years in males.

Figure 4 illustrates the age‐related increase in osteophyte scores in both sexes. Mild osteophytes were observed from approximately 1 year of age in males and females, whereas severe osteophytes appeared from around 8 years in females and 9 years in males. The lumbar region exhibited the highest cumulative burden, followed by the thoracic region, while cervical and sacral regions were less affected.

## 4 Discussion

The vertebral degeneration observed in *C. aethiops* followed a clear age‐related pattern, characterized by progressive bony changes and earlier involvement of the lumbar spine. Age was the primary determinant of vertebral degeneration across models. Body mass demonstrated independent associations in specific analyses, although its effect size was smaller than that of age. Sex showed no independent association with regional osteophyte presence, but exhibited a modest yet statistically significant association with total osteophyte burden in the adjusted Poisson model. This pattern closely parallels findings in other catarrhine primates, including *Macaca mulatta*, *M. fascicularis*, and *Papio* spp., in which osteophyte formation and intervertebral alterations increase with age and commonly affect thoracolumbar segments (Grynpas et al. 1993; Kramer et al. 2002; Nuckley et al. 2008). Importantly, our results extend this degenerative spectrum to a small‐ to medium‐bodied primate species ( ~ 3–6 kg), which is substantially lighter than the medium‐ to large‐bodied primates most frequently studied, such as *Macaca* spp. (~ 5–12 kg) and *Papio* spp. (~10–35 kg) (Grynpas et al. 1993; Kramer et al. 2002; Bailey et al. 2014). The greater body mass reported for these taxa is expected to impose higher axial loading on the vertebral column, influencing spinal biomechanics and potentially modulating interspecific patterns of vertebral degeneration (Nuckley et al. 2008; Bailey et al. 2014). Together, these findings highlight that comparable age‐related spinal changes occur across a broad range of body sizes, reinforcing age as the dominant driver of vertebral degeneration in catarrhine primates.

Vervet monkeys are a predominantly quadrupedal semi‐terrestrial NHP, alternating between arboreal and terrestrial substrates (Butynski and Jong 2022; Fleagle et al. 2024). Under laboratory conditions, however, reduced space and limited locomotor opportunities alter its natural behavioral repertoire, restricting individuals to chronic postural strain and biomechanical overload (Shively et al. 2012). Their semi‐terrestrial quadrupedal locomotion, alternating between the ground and trees, requires efficient support, especially of the lumbar spine and hindlimbs, to ensure stability and shock absorption during movement (Frye et al. 2021). As a form of adaptation to terrestrial and arboreal movement, greater osteomuscular robustness is observed in the hindlimbs according to morphometric analyses of *C. sabaeus*, in agreement with patterns of sexual dimorphism described for Old World primates (Plavcan 2001; Martonos et al. 2025). Recognizing and monitoring such musculoskeletal consequences is essential for refining husbandry practices, designing enclosures that promote species‐typical activity, and ultimately ensuring both animal welfare and the validity of biomedical research outcomes. Similar interactions between environmental constraints and aging have been reported in *Saimiri* spp., in which restricted housing conditions were associated with musculoskeletal and pelvic disorders, underscoring the role of husbandry in mitigating degenerative processes in captive primates (Pereira da Silva et al. 2021)

The high incidence of osteophytes observed in the lumbar region suggests a pattern of cumulative anteroposterior mechanical overload, possibly aggravated by less dynamic environments. These changes indicate progressive degeneration of the skeletal system from the cervical to the lumbar region, with potential clinical implications, such as joint stiffness, low back pain, and reduced mobility. Similar findings have also been described in *Macaca fascicularis*, in which osteophytosis and disc narrowing were associated with increased segmental stiffness and loss of dynamic spinal function (Nuckley et al. 2008).

Although osteophytes were the most frequent vertebral alteration, other osteopathies, such as discopathy, syndesmophytes, and scoliosis, appeared only sporadically. This pattern matches the expected degenerative sequence in primates, in which osteophytes arise early due to chronic mechanical remodeling of vertebral margins (Hernández‐Godínez et al. 2010; Kramer et al. 2002). Discopathy generally becomes radiographically evident only in later stages, after substantial structural deterioration of the intervertebral disc, as documented in *M. fascicularis*, *M. mulatta*, and, even, in humans (Nuckley et al. 2008; Bailey et al. 2014; Simmons 2016). Syndesmophytes and scoliosis, rare in our sample, are typically linked to inflammatory, congenital, or traumatic conditions rather than primary aging (Schmidt 2011; Grynpas et al. 1993). Together, these findings indicate that most animals were in early to intermediate stages of vertebral degeneration, with osteophytes serving as the predominant radiographic marker (Kim et al. 2011; Simmons 2016).

The handling environment can directly influence the musculoskeletal health of these animals. Prolonged exposure to artificial housing conditions may induce degenerative patterns in NHPs that closely resemble those in humans (Cooper et al. 2025). Confinement‐related factors, including reduced mobility, sedentary routines, and limited spatial complexity, appear to be critical determinants of vertebral degeneration. In great apes (chimpanzees), studies indicate that limited space and lack of stimuli in captivity reduce the diversity of natural movements, favoring repetitive postures and localized overload, which can accelerate degenerative processes (Cooper et al. 2025). In the context of reduced space, these results underscore the need for management strategies that mitigate such effects, primarily by increasing available space and implementing environmental enrichment designed to stimulate species‐typical locomotor behaviors and postural variation (Young et al. 2013; Lutz and Novak 2005). Incorporating these refinements not only promotes animal welfare but also enhances the reliability of musculoskeletal outcomes in biomedical research.

The high prevalence of osteopathic changes in the lumbar region observed in this study can be explained by biomechanical principles related to the stabilizing and load‐bearing function of this region. In humans, the lumbar spine acts to absorb impacts and redistribute forces applied to the trunk, being particularly susceptible to fatigue and chronic overload, as demonstrated by Barbosa and Gonçalves (2007) in electromyographic evaluations of the deep lumbar muscles. Although *C. aethiops* adopts a quadrupedal posture (Fleagle et al. 2024), the lumbar region of these primates continues to play a central role in supporting body weight and conducting mechanical forces between the trunk and hind limbs. As discussed by García Diez et al. (2024), biomechanical pressures imposed by postural and locomotor patterns throughout evolution have profoundly influenced the musculoskeletal architecture of primates, making regions such as the pelvis and spine vulnerable to disorders associated with chronic overload.

Consistent evidence from other Old World monkeys reinforces this interpretation. In *M. fascicularis*, lumbar vertebrae show marked age‐related degeneration, including osteophyte formation, disc space narrowing, and increased segmental stiffness (Nuckley et al. 2008; Bailey et al. 2014). Similar thoracolumbar patterns have been reported in *M. mulatta* and *Papio* spp., where vertebral osteoarthritis and disc degeneration accumulate with age and parallel changes described in humans (Kramer et al. 2002; Grynpas et al. 1993; Simmons 2016). These species differ in locomotor ecology, ranging from more arboreal to predominantly terrestrial, and encompass a wide spectrum of body sizes. Nevertheless, comparable age‐related degenerative patterns are observed in large, medium, and medium‐small cercopithecines, suggesting that body mass alone does not drive lumbar predominance. The lumbar susceptibility observed in *C. aethiops* therefore aligns with a conserved degenerative gradient across cercopithecines, highlighting shared biomechanical constraints among primates. Further studies, including smaller‐bodied species are needed to determine whether these patterns extend across the full primate size continuum.

Under human‐reared conditions, limited space and postural variability can intensify repetitive overload and reduce dynamic muscle stimulation, favoring the development of structural alterations, such as osteophyte formation. These bony projections arise as an adaptive response of bone tissue to chronic joint instability or mechanical wear (Pizetta et al. 2024). The analogy with human studies reinforces the interpretation that, regardless of the posture adopted, the lumbar region represents a zone of common biomechanical vulnerability among primates. However, in humans, the bipedal stance adds the cumulative weight of the upper body vertically over the lumbar spine, potentially increasing its biomechanical vulnerability (Lovejoy 2005).

Comparative studies in Old World primates consistently document age‐associated progression of vertebral degeneration in *C. sabaeus*, *Macaca* spp., and *Papio* spp. (Kramer et al. 2002; Nuckley et al. 2008; Simmons 2016; Martonos et al. 2025), highlighting the cumulative and progressive nature of spinal aging across cercopithecines. Rhesus macaques, in particular, exhibit a compressed but physiologically comparable aging trajectory to humans, with musculoskeletal decline paralleling broader systemic senescence (Chiou et al. 2020; Newman et al. 2023). Within this comparative framework, the lumbar‐dominant pattern of osteophyte formation observed in *C. aethiops* aligns with conserved degenerative pathways described in other catarrhines, supporting its relevance as a complementary model for primate spinal aging. Additionally, the lumbar region in humans seems to be susceptible to these changes because of the high mechanical load, responsible for absorbing impacts and redistributing forces along the axial axis (Barbosa and Gonçalves 2007). These findings reinforce the hypothesis that biological aging in *C. aethiops* plays a central role in the manifestation of these changes, highlighting the importance of clinical and management protocols aimed at elderly individuals under human care.

In terrestrial cercopithecines, such as those of the genera *Papio* and *Macaca*, it is already recognized that the pronograde posture, that is, the trunk remains approximately horizontal during locomotion and body weight is distributed across all four limbs is a common practice. When combined with repetitive movements on flat terrain, this generates concentrated mechanical stress points in the lumbar spine, favoring early degenerative changes (Schmidt 2011; Fleagle et al. 2024). Thus, the early appearance of osteophytes in *C. aethiop*s may be linked to a similar locomotor pattern, aggravated by inadequate handling or low movement variability.

Amaral Imbeloni et al. (2016) observed a progressive reduction in biochemical markers of bone remodeling, including procollagen type I N‐terminal propeptide (PINP), osteocalcin (OC), and parathyroid hormone (PTH), in aged *C. aethiops* individuals maintained under human care. This suggests that the development of degenerative changes in the spine, such as osteophyte formation, is more related to the aging of osteoarticular tissues and age‐associated mechanical loading, including body mass, than to sex differences. In the present study, although sex showed a statistically significant association with total osteophyte burden in the adjusted model, the magnitude of this effect was modest when compared with that of age and body mass.

This reinforces the hypothesis that spinal degeneration occurs predominantly because of cumulative processes associated with the aging of osteoarticular tissues. Structures such as intervertebral disks, cartilage, and ligaments undergo structural changes with advancing age, losing elasticity and functionality (Martonos et al. 2025). These changes, combined with chronic exposure to mechanical stress, favor the formation of osteophytes, indicating that age is the main factor involved in the genesis of the observed osteopathies, more so than sex or body mass.

Degenerative changes in the spine have been widely documented in captive primates, with significant radiographic and biomechanical impact. In *M. fascicularis*, Nuckley et al. (2008) observed osteophytes and narrowing of the intervertebral space in elderly individuals (mean age 22.3 years) housed individually throughout their lives. These changes were associated with increased disc stiffness, reduced energy absorption capacity, and loss of dynamic intervertebral disc function, characteristics consistent with advanced degeneration. Similarly, data from the present study suggest that a restrictive environment, including limited space and repetitive locomotor patterns, may favor chronic spinal overload in *C. aethiop*s, contributing to osteophyte formation.

Our findings show that vervet monkeys maintained under restricted conditions are prone to osteoarticular changes, highlighting the need for management strategies that actively promote locomotion and natural behaviors. Expanding physical space and implementing enrichment measures that stimulate three‐dimensional movement, exploration, and species‐typical activity can reduce postural strain and harmful repetitive patterns, thereby support musculoskeletal health and mitigating chronic stress. Such improvements enhance overall welfare while strengthening the validity of biomedical research outcomes. Beyond captive settings, these results are also relevant for free‐ranging primate populations, in which mobility limitations caused by vertebral degeneration may directly compromise individual performance, survival, and long‐term population viability, potentially exerting a greater impact than under human care.

## Author Contributions

**Camille Gabriela Ramos Portal:** investigation, writing – original draft, visualization. **Aline Amaral Imbeloni:** conceptualization, methodology, investigation, writing – original draft, visualization. **Sheila Canevese Rahal:** conceptualization, investigation, funding acquisition, writing – original draft, writing – review and editing, visualization, methodology. **Wellington Bandeira da Silva:** methodology, investigation, writing – original draft, visualization. **Washington Takashi Kano:** investigation, writing – original draft, visualization, writing – review and editing. **Seizo Yamashita:** investigation, visualization, writing – original draft. **Pedro Mayor:** methodology, investigation, writing – original draft, visualization, formal analysis. **Frederico Ozanan Barros Monteiro:** conceptualization, investigation, funding acquisition, writing – original draft, writing – review and editing, visualization, methodology, formal analysis, project administration, supervision.

## Conflicts of Interest

The authors declare no conflicts of interest.

## Supporting information


**Table S1:** Details of the model using Poisson GLM to verify the influence of sex, age and body mass on the Binomial GLMs for the presence of osteophytes in each region studied in the *Chlorocebus aethiops* (*n* = 70). **Table S2:** Details of the model using Poisson GLM to verify the influence of sex, age and body mass on the total osteophyte score in the *Chlorocebus aethiops* (*n* = 70).

## Data Availability

The data that support the findings of this study are available from the corresponding author upon reasonable request.
