# Supplementary material for: Radiographic Assessment of Spinal Degeneration in Vervet Monkeys (Chlorocebus aethiops): Prevalence, Patterns, and Relevance to Primate Aging Models
Source: Am J Primatol. 2026 Mar 23;88(3):e70136. doi: 10.1002/ajp.70136 (PMC13006768; doi:10.1002/ajp.70136)
Supplement: Supplementary file 1 — Table S1: Details of the model using Poisson GLM to verify the influence of sex, age and body mass on the Binomial GLMs for the presence of osteophytes in each region studied in the Chlorocebus aethiops (n = 70). Table S2: Details of the model using Poisson GLM to verify the influence of sex, age and body mass on the total osteophyte score in the Chlorocebus aethiops (n = 70). [file AJP-88-e70136-s001.docx]

**Table S1.** Details of the model using Poisson GLM to verify the influence of sex, age and body mass on the Binomial GLMs for the presence of osteophytes in each region studied in the *Chlorocebus aethiops* (n = 70). Outcome coded as 1 = presence of osteophytes (any grade), 0 = absent. Sex_F = 1 for females and 0 for males.

| **Region Parameter** | **Coef. (β)** | **SE** | **z** | **p** |
| --- | --- | --- | --- | --- |
| **Cervical** | | | | |
| Intercept | -8.504 | 3.127 | -2.72 | 0.007 |
| Sex_F | 1.177 | 1.040 | 1.13 | 0.258 |
| Age (years) | 0.273 | 0.072 | 3.78 | <0.001 |
| Body mass (kg) | 1.123 | 0.570 | 1.97 | 0.049 |
| **Thoracic** | | | | |
| Intercept | -7.389 | 2.516 | -2.94 | 0.003 |
| Sex_F | 1.784 | 0.943 | 1.89 | 0.058 |
| Age (years) | 0.178 | 0.051 | 3.49 | <0.001 |
| Body mass (kg) | 0.856 | 0.468 | 1.83 | 0.068 |
| **Lumbar** | | | | |
| Intercept | -2.761 | 1.550 | -1.78 | 0.075 |
| Sex_F | 1.353 | 0.872 | 1.55 | 0.121 |
| Age (years) | 0.148 | 0.070 | 2.11 | 0.034 |
| Body mass (kg) | 0.525 | 0.411 | 1.28 | 0.202 |
| **Any region** | | | | |
| Intercept | -3.246 | 1.619 | -2.01 | 0.045 |
| Sex_F | 1.391 | 0.892 | 1.56 | 0.119 |
| Age (years) | 0.117 | 0.068 | 1.74 | 0.082 |
| Body mass (kg) | 0.773 | 0.441 | 1.75 | 0.080 |

**Table S2.** Details of the model using Poisson GLM to verify the influence of sex, age and body mass on the total osteophyte score in the *Chlorocebus aethiops* (n = 70). Outcome: total osteophyte score per animal. Sex_F = 1 for females and 0 for males.

| **Parameter** | **Coef. (β)** | **SE** | **z** | **p** |
| --- | --- | --- | --- | --- |
| Intercept | -1.450 | 0.509 | -2.85 | 0.004 |
| Sex_F | 0.461 | 0.234 | 1.97 | 0.049 |
| Age (years) | 0.069 | 0.012 | 5.92 | <0.001 |
| Body mass (kg) | 0.254 | 0.103 | 2.47 | 0.014 |
